# Controlling Cellular P-TEFb Activity by the HIV-1 Transcriptional Transactivator Tat

**DOI:** 10.1371/journal.ppat.1001152

**Published:** 2010-10-14

**Authors:** Lisa Muniz, Sylvain Egloff, Bettina Ughy, Beáta E. Jády, Tamás Kiss

**Affiliations:** 1 Laboratoire de Biologie Moléculaire Eucaryote du CNRS, UMR5099, IFR109 CNRS, Université Paul Sabatier, Toulouse, France; 2 Biological Research Centre, Hungarian Academy of Sciences, Szeged, Hungary; Duke University Medical Center, United States of America

## Abstract

The human immunodeficiency virus 1 (HIV-1) transcriptional transactivator (Tat) is essential for synthesis of full-length transcripts from the integrated viral genome by RNA polymerase II (Pol II). Tat recruits the host positive transcription elongation factor b (P-TEFb) to the HIV-1 promoter through binding to the transactivator RNA (TAR) at the 5′-end of the nascent HIV transcript. P-TEFb is a general Pol II transcription factor; its cellular activity is controlled by the 7SK small nuclear RNA (snRNA) and the HEXIM1 protein, which sequester P-TEFb into transcriptionally inactive 7SK/HEXIM/P-TEFb snRNP. Besides targeting P-TEFb to HIV transcription, Tat also increases the nuclear level of active P-TEFb through promoting its dissociation from the 7SK/HEXIM/P-TEFb RNP by an unclear mechanism. In this study, by using *in vitro* and *in vivo* RNA-protein binding assays, we demonstrate that HIV-1 Tat binds with high specificity and efficiency to an evolutionarily highly conserved stem-bulge-stem motif of the 5′-hairpin of human 7SK snRNA. The newly discovered Tat-binding motif of 7SK is structurally and functionally indistinguishable from the extensively characterized Tat-binding site of HIV TAR and importantly, it is imbedded in the HEXIM-binding elements of 7SK snRNA. We show that Tat efficiently replaces HEXIM1 on the 7SK snRNA *in vivo* and therefore, it promotes the disassembly of the 7SK/HEXIM/P-TEFb negative transcriptional regulatory snRNP to augment the nuclear level of active P-TEFb. This is the first demonstration that HIV-1 specifically targets an important cellular regulatory RNA, most probably to promote viral transcription and replication. Demonstration that the human 7SK snRNA carries a TAR RNA-like Tat-binding element that is essential for the normal transcriptional regulatory function of 7SK questions the viability of HIV therapeutic approaches based on small drugs blocking the Tat-binding site of HIV TAR.

## Author Summary

Expression and replication of the human immunodeficiency virus (HIV) is supported by the viral transcriptional transactivator (Tat) that recruits the host positive transcription elongation factor b (P-TEFb) to the promoter of the integrated viral genome. Here, we demonstrate that HIV Tat specifically and efficiently binds to the host 7SK small nuclear RNA (snRNA) that is a negative regulator of P-TEFb. Although HIV Tat has been reported to interact with a plethora of host factors, our results indicate that the 7SK transcriptional regulatory snRNA is a major and important cellular target of HIV Tat. We demonstrate that binding of Tat to the 7SK snRNA disrupts the 7SK-P-TEFb negative transcriptional regulatory complex and releases active P-TEFb. Thus, we propose that Tat not only targets P-TEFb for HIV transcription, but also modulates the nuclear level of active P-TEFb in HIV-infected cells.

## Introduction

Synthesis of mRNAs by Pol II is tightly controlled at the step of transcription elongation by the positive transcription elongation factor b (P-TEFb) that is a cyclin-dependent kinase composed of Cdk9 and cyclin T1 (CycT1) [1], [2], [3], [4], [5]. After transcription initiation and promoter clearance, Pol II is arrested by the negative elongation factor (NELF) and the DRB sensitivity-inducing factor (DSIF). To restore productive Pol II elongation, P-TEFb phosphorylates NELF, DSIF and the heptapeptide repeats (YSPTSPS) in the C-terminal domain (CTD) of Pol II at serine 2. P-TEFb is a general transcription factor that is required for efficient expression of most protein-coding genes as well as for production of full-length transcripts from the integrated HIV-1 genome [6], [7].

In the nuclei of HeLa cells, about half of P-TEFb forms a kinase-inactive ribonucleoprotein (RNP) with the 7SK snRNA [8], [9]. The 7SK/P-TEFb snRNP also contains the hexamethylene bisacetamide (HMBA)-inducible protein HEXIM1 and less often, HEXIM2 [10], [11], [12], [13], the La-related protein Larp7 [14], [15], [16] and the methylphosphate capping enzyme MePCE [17], [18]. While Larp7 and MePCE bind stably to and provide stability for 7SK snRNA, P-TEFb and HEXIM1/2 show a dynamic, transcription-dependent association with 7SK. Blocking of Pol II transcription induces dissociation of P-TEFb and HEXIM proteins from the 7SK snRNP to increase the nuclear level of active P-TEFb [8], [9], [10], [11]. On the contrary, inhibition of cell growth shifts P-TEFb from active to inactive 7SK-associated complexes [19], [20]. Thus, the 7SK snRNA and HEXIM1/2 proteins function as key regulators of Pol II transcription through controlling the nuclear activity of P-TEFb. Malfunction of the 7SK–P-TEFb regulatory machine that abnormally increases P-TEFb activity can lead to development of cardiac hypertrophy or to malignant transformation of the cell [16], [21].

The human 7SK is a 331 nt-long Pol III-transcribed abundant snRNA [22]. P-TEFb is tethered to 7SK through interacting with HEXIM1 and HEXIM2 that directly bind to the 5′ hairpin of 7SK snRNA in the forms of homo- or heterodimers [11], [12], [13], [23], [24], [25], [26], [27]. HEXIM proteins interact with two copies of P-TEFb and inhibit their protein kinase activity strictly in a 7SK snRNA-dependent manner [11], [27]. Binding of 7SK to the positively charged RNA-binding motif of HEXIM1/2 enables the acidic P-TEFb-binding domain of HEXIM1/2 to interact with CycT1 [28]. *In vivo* docking and inactivation of P-TEFb by the 7SK snRNP also requires the binding of CycT1, either directly or indirectly, to the 3′ hairpin of 7SK snRNA [26].

Transcription initiated from the long terminal repeat (LTR) promoter of the integrated HIV-1 genome is controlled predominantly at the level of elongation [1], [29], [30]. The processivity of HIV transcription depends on the viral transactivator Tat that recruits P-TEFb to the stalled Pol II [31], [32]. To capture P-TEFb, the activation domain of Tat associates with CycT1 and its RNA-binding motifs binds to the transactivation response element, TAR, an RNA hairpin at the 5′ end of the nascent HIV LTR transcript [33], [34], [35], [36]. Besides tethering P-TEFb to the TAR RNA, recent studies demonstrated that Tat also promotes the disassembly of the 7SK/HEXIM/P-TEFb snRNP to increase the nuclear level of active P-TEFb [37], [38]. Indeed, efficient transcription from the HIV LTR promoter requires considerably higher P-TEFb activity than that is needed for cellular mRNA production and host cell viability [6], [31], [39], [40], [41].

The molecular mechanism of Tat-mediated regulation of nuclear P-TEFb activity is unclear. Tat has been proposed to compete with HEXIM to displace it either from the CycT1 subunit of P-TEFb or from the 7SK snRNA [37], [38]. In this study, we demonstrate that in HIV-infected cells, the 7SK transcriptional regulatory snRNA is the major RNA target of the accumulating Tat protein. HIV Tat binds with high specificity and efficiency to an evolutionarily highly conserved TAR RNA-like stem-bulge-stem motif of the 5′ hairpin of 7SK snRNA. We demonstrate that the newly discovered Tat-binding site of 7SK is imbedded in the HEXIM-binding elements of 7SK and therefore, Tat promotes disassembly of the 7SK/HEXIM/P-TEFb negative transcriptional regulatory snRNP through displacing the HEXIM homodimer from the 7SK snRNA.

## Results

### HIV Tat specifically associates with 7SK snRNA *in vivo*

**Figure 1:**
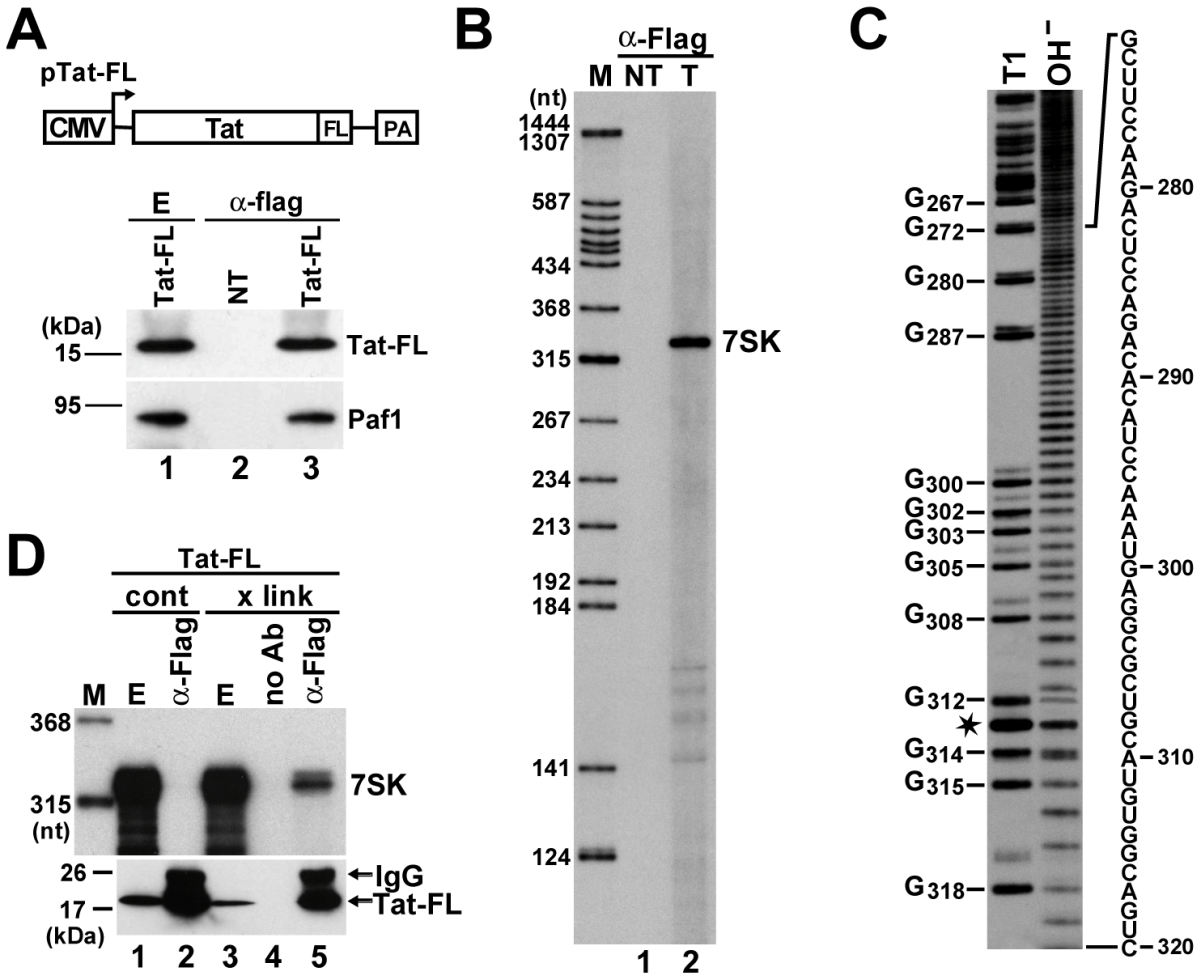
*In vivo* association of HIV Tat with 7SK. A. Transient expression of Tat-FL in HeLa cells. Schematic structure of the pTat-FL expression construct is shown. The cytomegalovirus promoter (CMV) and the polyadenylation region (PA) are indicated. Tat-FL was immunoprecipitated (α-Flag) from extracts (E) prepared from transfected or non-transfected (NT) cells. Distribution of Tat-FL and Paf1 was monitored by Western blot analysis. B. Detection of Tat-associated HeLa RNAs. RNAs co-precipitated with Tat-FL (T) were labeled *in vitro* and separated on a 6% sequencing gel. Lanes NT and M, control IP from non-transfected cells and molecular size markers. C. RNA G-tracking. The Tat-associated RNA was partially digested with RNase T1 or moderately hydrolyzed with formamide (OH^−^) and analyzed on a 6% gel. G residues and their positions in the human 7SK snRNA sequence are indicated. Asterisk indicates a fragile U residue. D. *In vivo* cross-linking of Tat-FL and 7SK. HeLa cells expressing Tat-FL were treated (x link) or not treated (cont) with formaldehyde before extract (E) preparation. Tat-FL was immunoprecipitated (α-Flag) or mock-precipitated (no Ab) under stringent conditions. Distributions of Tat-FL and 7SK snRNA were monitored by Western blot analysis and RNase A/T1 mapping, respectively.

To test whether Tat can interact with 7SK snRNA in living cells, Flag-tagged Tat (Tat-FL) was transiently expressed in human HeLa cells and recovered by immunoprecipitation (IP) with an anti-Flag antibody (Figure 1A). As demonstrated by Western blot analysis, the expressed Tat-FL protein interacted with the Paf1 component of the recently reported Tat/P-TEFb-associated elongation complex, indicating that it was functionally active [42]. HeLa RNAs co-precipitated with Tat-FL were 3′ end-labeled with [5′-^32^P]pCp and T4 RNA ligase and analyzed on a denaturing gel (Figure 1B, lane 2). Autoradiography revealed that Tat-FL bound to a unique RNA with an electrophoretic mobility corresponding to the 331 nt-long human 7SK snRNA. To determine unequivocally its identity, the terminally labeled Tat-associated RNA was partially digested with the G-specific endoribonuclease T1 or it was moderately hydrolyzed with alkali before fractionation on a sequencing gel (Figure 1C). Distribution of the G residues in the 3′-terminal part of the Tat-associated HeLa RNA perfectly matched with the nucleotide sequence of human 7SK snRNA. The faint band duplications above the RNase T1 digestion products indicate sequence heterogeneity at the 3′ end of 7SK snRNA [43].

To rule out that Tat-FL associated with 7SK in the cell extract, we performed *in vivo* cross-linking experiments (Figure 1D). HeLa cells expressing Tat-FL were treated with formaldehyde and after extract preparation, Tat-FL was immunoprecipitated under highly stringent conditions [44]. RNase A/T1 protection analysis confirmed that 7SK snRNA was efficiently cross-linked to Tat-FL *in vivo* (lane 5). In control IPs performed in the absence of antibody (lane 4) or from non-cross-linked cell extract (lane 2), no 7SK-Tat-FL interaction was detected under the applied harsh wash conditions. We concluded that Tat specifically and most probably, directly interacts with 7SK snRNA in living cells.

### The 5′ hairpin of human 7SK snRNA carries a conserved Tat-binding element

**Figure 2:**
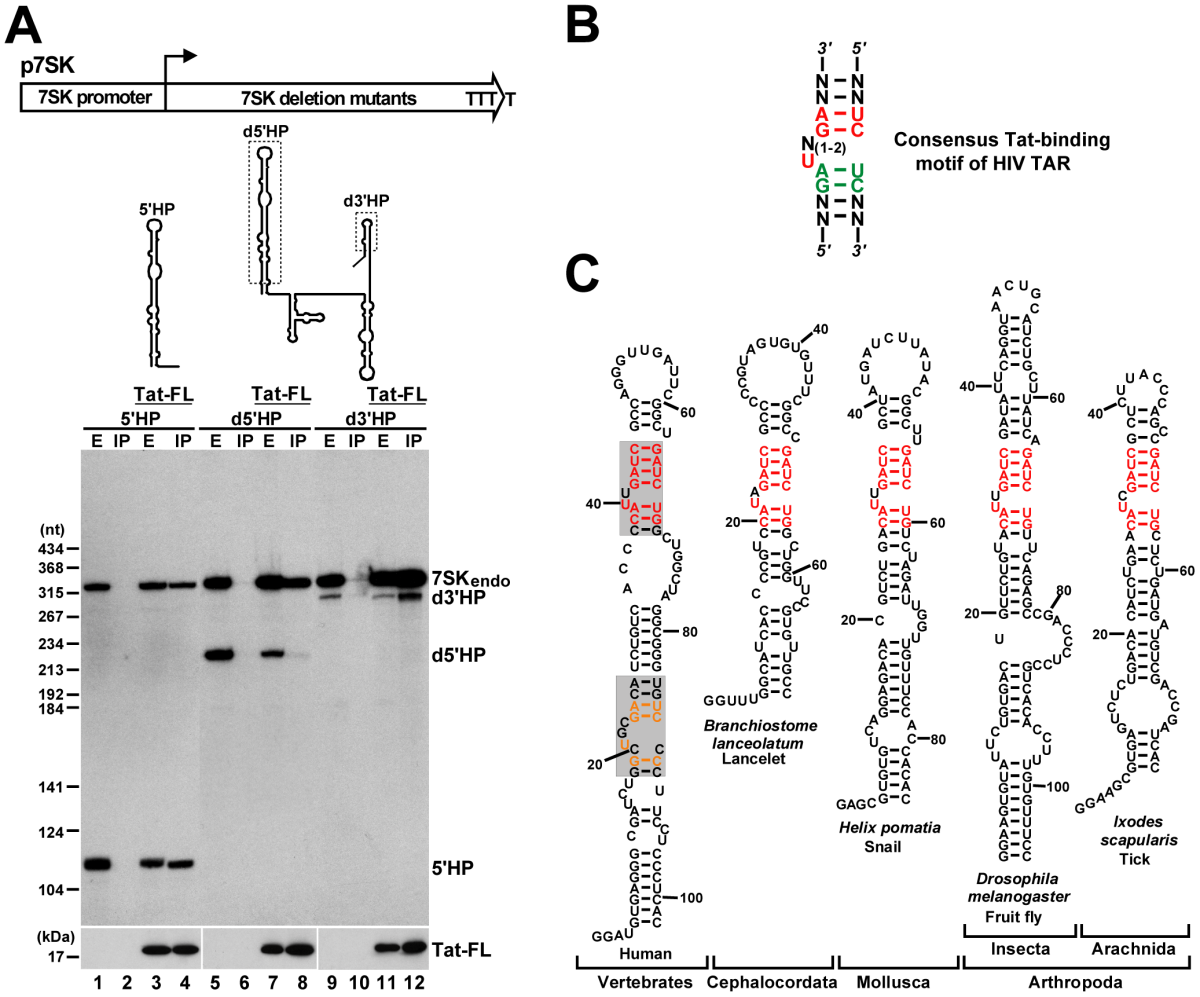
Identification of putative Tat-binding motifs in 7SK snRNA. A. Tat binds to the 5′ hairpin of 7SK. Schematic structures of the p7SK expression construct and the expressed truncated 7SK RNAs are shown. Dashed boxes indicate deletions. Pol III transcription of the *7SK* gene terminates within four consecutive T residues. HeLa cells were transfected with the indicated expression plasmids. After extract (E) preparation, Tat-FL was immunoprecipitated (IP). Distribution of the endogenous and transiently expressed 7SK RNAs and Tat-FL was monitored by Western and Northern blotting. B. Consensus structure of the minimal Tat-binding motif of HIV TAR. Nucleotides with essential and moderate contribution to Tat binding are in red and green, respectively. C. Phylogenetic comparison of the 5′ hairpins of 7SK snRNAs. The sequences of lancelet, snail, fruit fly and tick 7SK snRNAs have been published [65], [66]. The potential Tat-binding motifs of human 7SK snRNA are shaded. The evolutionarily invariant nucleotides are in red. Nucleotides common to the putative proximal Tat-binding motif of 7SK and the Tat-binding site of HIV TAR are in orange.

To define the region of 7SK snRNA which interacts with Tat, we assayed the *in vivo* interaction of transiently expressed truncated 7SK RNAs with Tat-FL by IP with an anti-Flag antibody followed by Northern blot analysis (Figure 2A). Co-precipitation of the endogenous HeLa 7SK snRNA with Tat-FL provided a positive control for each IP reaction. Removal of the 3′ hairpin of 7SK, although largely compromised the stability of the truncated d3′HP RNA (lanes 9 and 11), failed to prevent its association with Tat-FL (lane 12). In contrast, deletion of the 5′ hairpin eliminated the interaction of d5′HP RNA with Tat-FL (lane 8). Finally, the 5′HP RNA that represented the 5′ hairpin of 7SK efficiently interacted with Tat-FL (lane 4), demonstrating that HIV Tat binds to the 5′ hairpin of 7SK snRNA.

Tat recognizes an internal stem-bulge-stem motif of HIV TAR RNA (Figure 2B). The first uridine in the bulge and the G-C and A-U base-pairs in the upper stem are indispensable for Tat binding [45], [46], [47], [48]. A structural rearrangement of the bulge loop provides the specificity for the Tat-TAR interaction. The bulged U forms a base-triple interaction with the A-U base-pair that stabilizes the association of the lower G residue in the major grove with an arginine of Tat [49], [50]. We noticed that the 5′ hairpin of human 7SK snRNA carries two internal segments, G18-A27/U84-C90 and C37–C45/G64–G70, which are highly reminiscent of the consensus minimal structure of the Tat-binding element of HIV TAR (Figure 2C, shaded boxes). The putative distal (upper) Tat-binding element of human 7SK shows a striking conservation in all known 7SK snRNAs derived from phylogenetically distant species.

**Figure 3:**
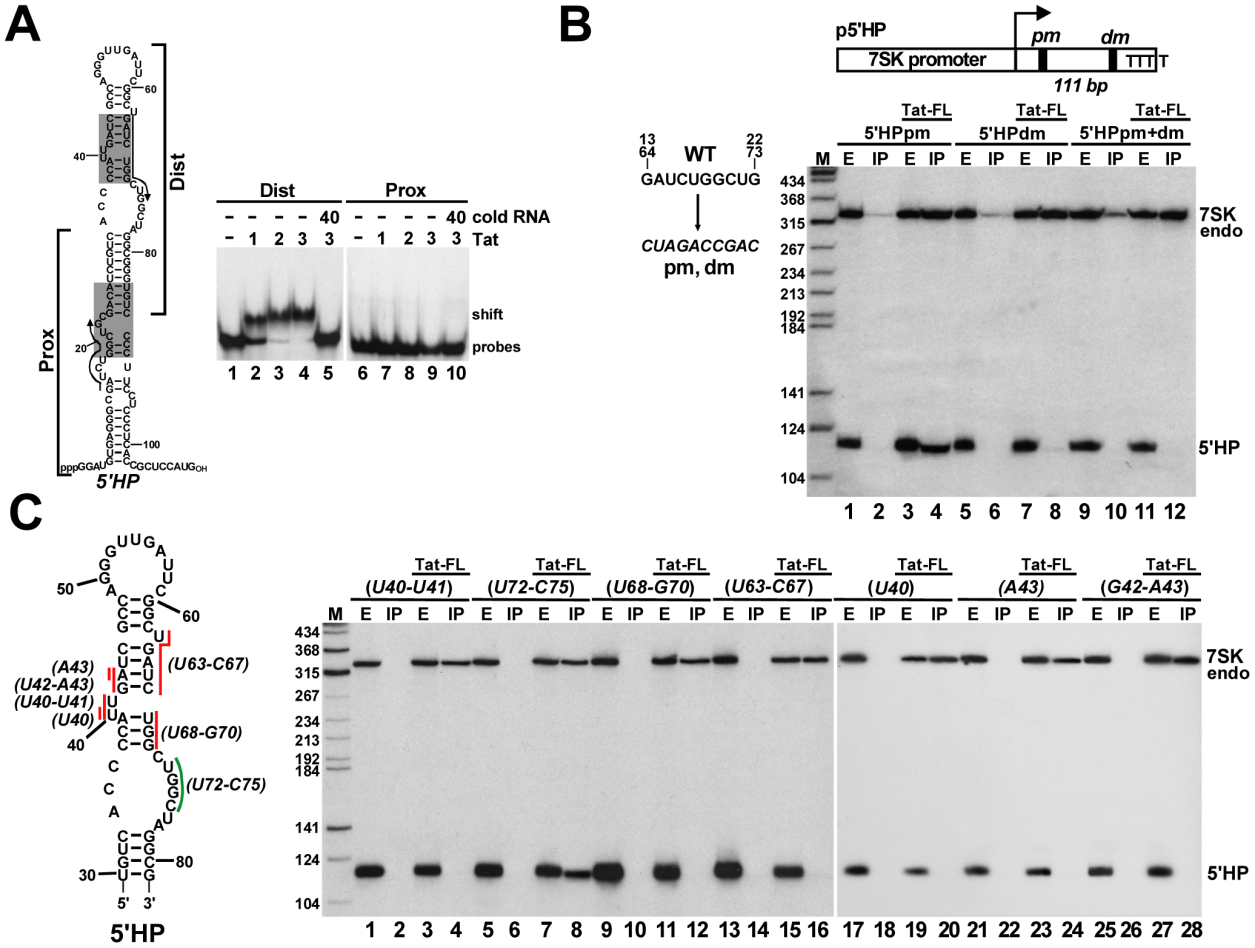
Characterization of the Tat-binding element of human 7SK snRNA. A. Tat binds to the distal part of the 5′ hairpin of 7SK. About 2 fmol of ^32^P-labeled RNA representing the distal (Dist) or proximal (Prox) part of the 7SK 5′ hairpin was incubated with the indicated amount (fmol) of Tat(38–72) oligopeptide and analyzed on a 4% native gel. B. The distal Tat-binding motif of 7SK directs *in vivo* binding of Tat. The 5′ hairpin of 7SK (5′HP) carrying the pm and/or dm mutations was co-expressed with Tat-FL and their interaction was tested by co-IP and Northern blotting. Structure of the p5′HP expression plasmid with the pm and dm mutations and the expected length of the 5′HP RNA is shown. C. *In vivo* association of Tat with mutant 7SK 5′ hairpin RNAs. Nucleotides indicated by red (essential) or green (dispensable) lines were replaced with complementary nucleotides in the p5′HP expression plasmid. Tat-FL and the mutant 5′HP RNAs were co-expressed in HeLa cells and their interactions were tested.

To test whether the newly detected potential Tat-binding motifs of human 7SK snRNA can interact with Tat, we performed electrophoretic mobility shift assays using *in vitro* synthesized probe RNAs representing either the distal (Dist) or the proximal (Prox) parts of the 5′ hairpin of 7SK and a Tat-derived oligopeptide, Tat(38–72) [51] (Figure 3A). The Tat peptide efficiently bound to the distal part of the 5′ hairpin of 7SK (lanes 2–4), but failed to associate with its proximal part (lane 7–9). Administration of cold Dist RNA abolished association of the Tat oligopeptide with 7SK sequences, confirming that Tat binds specifically to the distal part of the 5′ hairpin of human 7SK snRNA (lane 5).

The 5′ hairpin of human 7SK contains two 10 nt-long perfect repeats (G13–G22 and G64–G73) which overlap the distal and proximal putative Tat-binding elements (Figure 3A, indicated by arrows). The wild-type GAUCUGGCUG repeat sequences were replaced with complementary sequences in the p5′HP expression plasmid (Figure 3B). The mutant 5′HPdm (distal mutant) and 5′HPpm (proximal mutant) RNAs were transiently expressed in HeLa cells and their association with the co-expressed Tat-FL protein was tested. Northern blot analysis demonstrated that Tat-FL interacted with 5′HPpm RNA (lane 4), but it failed to associate with the 5′HPdm and the double mutant 5′HPpm+dm RNAs (lanes 8 and 12), providing strong support to the notion that Tat interacts with the distal Tat-binding motif of human 7SK snRNA.

To confirm that Tat binds to the C37–C45/G64–G70 TAR RNA-like motif of human 7SK, a series of mutant 5′HP RNAs were transiently expressed and their association with Tat-FL was examined (Figure 3C). Substitution of the U40, U40–U41 bulge nucleotides or the A43, G42-A43, U63-C67 and U68-G70 stem nucleotides for complementary sequences fully abolished the *in vivo* association of the expressed mutant 5′HP RNAs with Tat-FL (lanes 4, 12, 16, 20, 24 and 28). In contrast, substitution of the U72-C75 nucleotides failed to interfere with Tat binding (lane 8). Likewise, replacing the U63 bulge nucleotide, the G60-C62 stem or the A49-C59 loop sequences with complementary nucleotides had no effect on *in vivo* Tat binding (data not shown). We concluded that Tat binds to the evolutionarily conserved C37–C45/G64–G70 motif of 7SK that is structurally indistinguishable from the Tat-binding element of HIV TAR.

### The 5′ hairpin of human 7SK snRNA carries two HEXIM-binding sites

**Figure 4:**
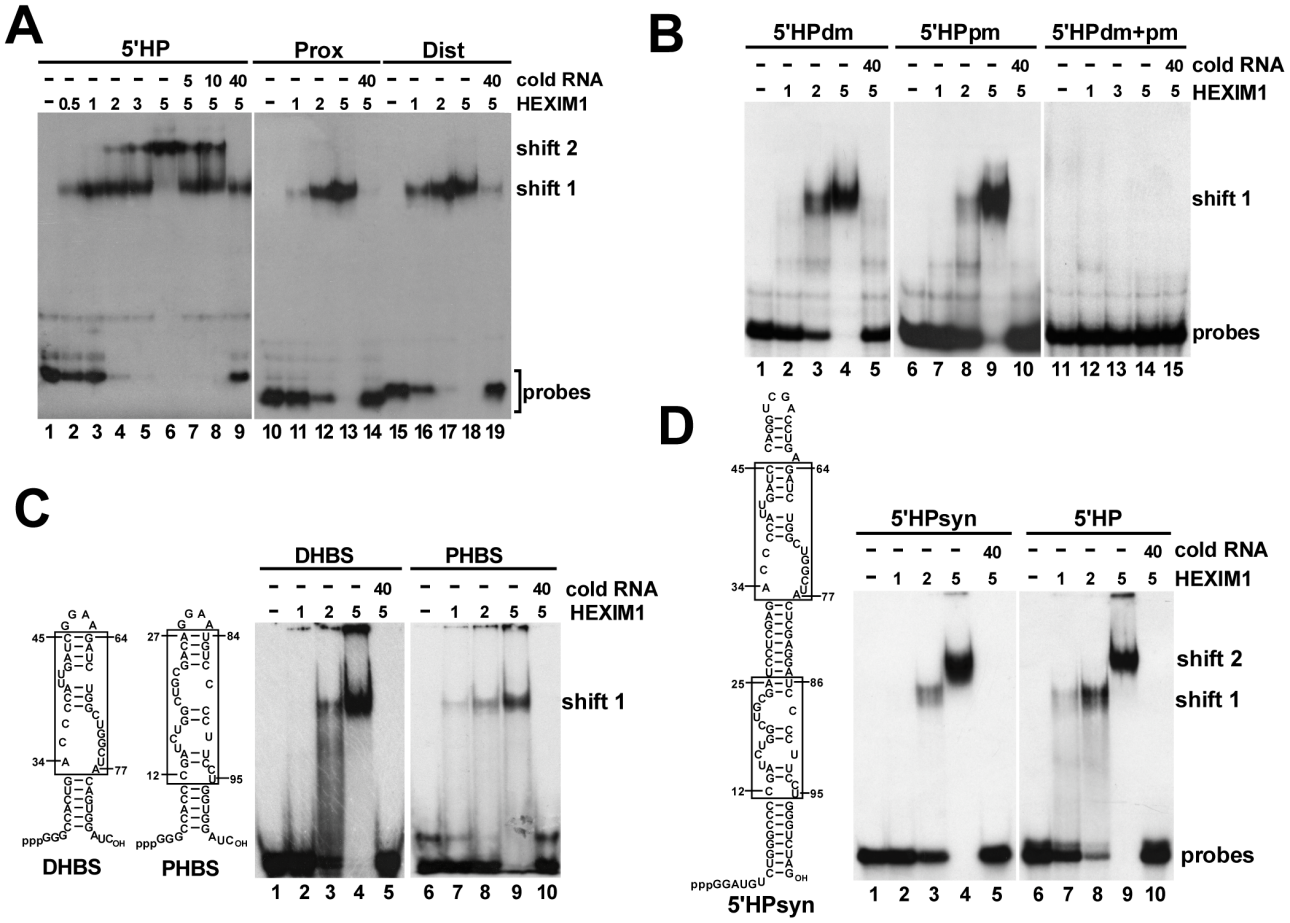
The 5′ hairpin of human 7SK snRNA contains two HEXIM-binding sites. A. Detection of HEXIM-binding sites by mobility shift assays. About 2 fmol of *in vitro* synthesized probe RNAs representing the entire (5′HP) or the distal (Dist) and proximal (Prox) parts of the 5′ hairpin of human 7SK were incubated with increasing amounts (fmol) of recombinant HEXIM1 and analyzed on a 5% native gel. Appropriate cold RNAs were used as specific competitors. B. *In vitro* interaction of mutant 7SK 5′ hairpin RNAs (5′HPdm, 5′HPpm, 5′HPdm+pm) with HEXIM1. Complexes were analyzed on a 4% gel. C. The minimal HEXIM1-binding elements of 7SK. The *in vitro* HEXIM-binding capacity of a distal (DHBS) and proximal (PHBS) fragment of the 7SK 5′ hairpin was tested by mobility shift assay on a 4% gel. Sequences derived from wild-type 7SK are boxed. D. Gelshift analysis of an artificial hairpin RNA (5′HPsyn) carrying the distal and proximal HEXIM-binding motifs of 7SK. Sequences originated from the human 7SK snRNA are boxed.

The strong evolutionarily conservation of the newly identified Tat-binding site of human 7SK suggests that this element plays an important role in the normal function of 7SK snRNA (Figure 2C). The positively charged arginine-rich TAR-recognition motif of HIV Tat shows strong similarity to the N-terminal part of the 7SK-binding motif of HEXIM proteins derived from evolutionarily distant species (Figure S1) [52]. This suggests that Tat and HEXIM recognize similar, if not identical, target motif(s) in the 5′ hairpin of 7SK. HEXIM1 has been reported to be a promiscuous double-stranded RNA-binding protein that binds to 7SK between nucleotides 10 to 48 in a sequence-independent manner [53]. In contrast, we had earlier observed that in HeLa cells HEXIM1 binds to the distal part of the 5′ hairpin of 7SK with high specificity [26]. To clarify these inconsistencies and to define the precise binding site of HEXIM, we performed electrophoretic mobility shift assays (Figure 4A). When the entire 5′ hairpin of 7SK (5′HP) was incubated with increasing amounts of recombinant HEXIM1, two 5′HP-HEXIM1 complexes, indicated as shift 1 and 2, were detected on a native gel (lanes 2–6). In the presence of about two-fold excess of HEXIM1 only the upper low-mobility complex (shift 2) was formed (lane 6). Administration of cold 5′HP RNA inhibited 5′HP-HEXIM1 complex formation (lanes 7–9), indicating that in accordance with previous reports, the 5′ hairpin of 7SK specifically associates with two molecules of HEXIM1 [13], [24], [25].

When probe RNAs representing the proximal (Prox) and distal (Dist) regions of the 5′ hairpin of 7SK were incubated with HEXIM1, HEXIM1 specifically associated with both RNAs (lanes 11–13 and lanes 16–18). The resulting Dist-HEXIM1 and Prox-HEXIM1 complexes co-migrated with the high-mobility complex (shift1) formed by the full-length 5′ hairpin and HEXIM1. Importantly, neither the Dist nor the Prox probe RNA formed the large low-mobility complex (shift 2) with HEXIM1, demonstrating that the 5′ hairpin of 7SK contains two structurally and functionally independent HEXIM-binding sites each recruiting one HEXIM molecule in an independent fashion *in vitro*.

We tested whether the newly defined distal Tat-binding element and the proximal Tat-binding-like motif of the 5′ hairpin of 7SK are essential for HEXIM1 binding. Mutant 5′ hairpin RNAs, 5′HPdm and 5′HPpm (see Figure 3), were incubated with HEXIM1 and the resulting complexes were analyzed on a native gel (Figure 4B). Both 5′HPdm and 5′HPpm RNAs formed only the high-mobility complex with HEXIM1 (shift 1), indicating that they bind only one copy of HEXIM1 (lanes 3–4 and 8–9). As expected, the double-mutant 5′HPpm+dm RNA was inactive in HEXIM-binding (lanes 12–14). These results confirmed that the newly identified Tat-binding motif in the distal part and the Tat-binding-like element in the proximal part of the 7SK 5′ hairpin function in HEXIM-binding.

To further delimit the snRNA elements directing *in vitro* HEXIM-binding, the distal A34-C45/G64-A77 and proximal C12-A27/U84-U95 fragments of 7SK were topped with GGAA tetraloops and stabilized with artificial basal stems (Figure 4C). The resulting distal and proximal HEXIM-binding site (DHBS and PHBS) RNAs specifically associated with HEXIM1 (lanes 4 and 9). Any further truncations or sequence alterations abolished the HEXIM-binding capacity of the DHBS and PHBS RNAs, indicating that the A34-C45/G64-A77 and C12-A27/U84-U95 internal segments of the 5′ hairpin of 7SK contain the minimal sequence and structural information required for *in vitro* recognition by HEXIM1 (data not shown). This conclusion was further corroborated by demonstration that similarly to the control wild-type 5′ hairpin (Figure 4D, lane 9), an artificial hairpin RNA (5′HPsyn) encompassing the C12-A25/U86-U95 and A34-C45/G64-A77 fragments of human 7SK was capable of binding two HEXIM1 molecules (lane 4).

### The distal and proximal HEXIM-binding sites of 7SK work in an interdependent fashion *in vivo*

**Figure 5:**
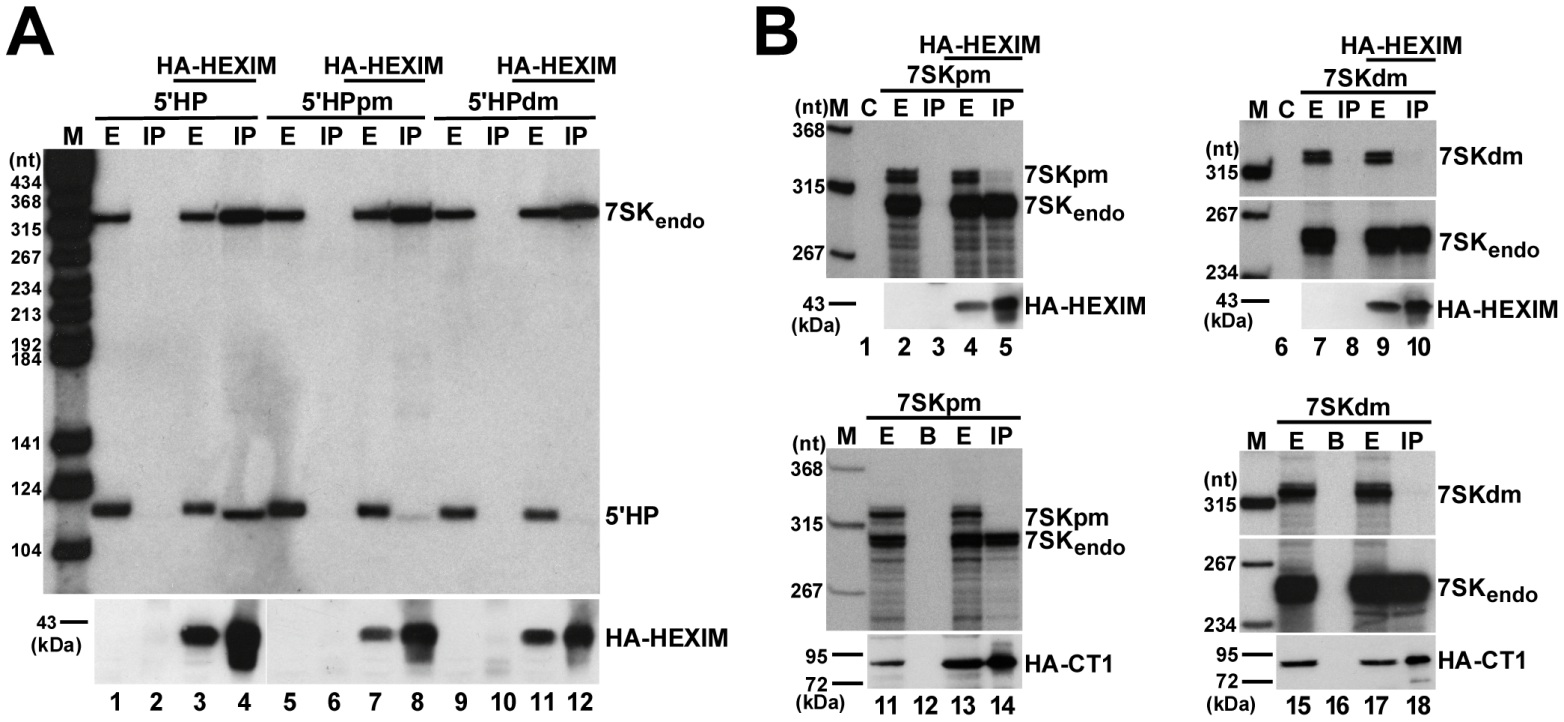
*In vivo* binding of HEXIM1 to 7SK RNA. A. Interaction of HEXIM1 with mutant 7SK 5′ hairpin RNAs. HA-HEXIM1 was immunoprecipitated (IP) from extracts (E) prepared from HeLa cells also expressing 5′HP, 5′HPpm or 5′HPdm RNAs. Interaction of HA-HEXIM1 with the endogenous 7SK and the ectopically expressed 5′HP, 5′HPpm, 5′HPdm RNAs was monitored by Northern blot analyses. B. Interaction of HEXIM1 and P-TEFb with mutant 7SK snRNAs. 7SKpm and 7SKdm RNAs were expressed in HeLa cells together with HA-HEXIM1 (lanes 1–10) or in G3H cells accumulating HA-CycT1 (lanes 11–18). After IP, recovery of HA-HEXIM1 and HA-CycT1 was confirmed by Western blot analysis and co-precipitation of the endogenous and ectopically expressed 7SK RNAs was determined by RNase A/T1 mapping. Lane C, control mapping with *E. coli* tRNA. Lane B, control IP with beads alone.

Our *in vitro* binding studies revealed that the 5′ hairpin of human 7SK snRNA carries two structurally and functionally independent HEXIM-binding sites. However, we had earlier observed that mutations predicted to disrupt the distal HEXIM-binding element of the 5′ hairpin fully abolished the *in vivo* HEXIM-binding capacity of 7SK snRNA [26]. A possible interpretation of these contradictory results could be that *in vivo* the two HEXIM-binding sites of 7SK function in an interdependent way. To test this assumption, we investigated the *in vivo* HEXIM-binding ability of the mutant 5′HPpm and 5′HPdm RNAs which still bind one copy of HEXIM1 under *in vitro* conditions (see Figure 4B). The 5′HPpm and 5′HPdm RNAs were transiently expressed in HeLa cells and their association with a co-expressed HA-tagged HEXIM1 was monitored (Figure 5A). The ectopically expressed HA-HEXIM1 protein efficiently associated with the endogenous HeLa 7SK snRNA (lanes 4, 8 and 12) and the transiently expressed wild-type 5′HP RNA (lane 4), but it showed no association with the mutant 5′HPdm and 5′HPpm RNAs (lanes 8 and 12), indicating that both HEXIM1-binding sites are required for *in vivo* recruitment of HEXIM1.

Next, we assayed the *in vivo* interaction of HA-HEXIM1 with transiently expressed full-length 7SK RNAs which, similarly to the 5′HPdm and 5′HPpm RNAs, carried the pm or dm sequence alterations (Figure 5B). In order to distinguish between the ectopically expressed mutant and the endogenous wild-type 7SK RNAs, RNase A/T1 mappings were performed using antisense probe RNAs specific for the mutant 7SKdm and 7SKpm snRNAs. In contrast to the endogenous 7SK snRNA, the transiently expressed 7SKdm and 7SKpm RNAs failed to efficiently associate with HA-HEXIM1 *in vivo* (lanes 5 and 10). Since docking of HEXIM1 is a prerequisite for P-TEFb binding, neither 7SKdm nor 7SKpm associated with P-TEFb, as demonstrated by co-IPs with HA-tagged CycT1 (lanes 14 and 18). The finding that disruption of either the distal or the proximal HEXIM-binding motif of 7SK abolishes the recruitment of both HEXIM and P-TEFb demonstrates that under *in vivo* conditions the distal and proximal HEXIM-binding sites of 7SK recruit two copies of HEXIM1 in a tightly interdependent manner.

### Tat competes with HEXIM1 for 7SK snRNA binding

**Figure 6:**
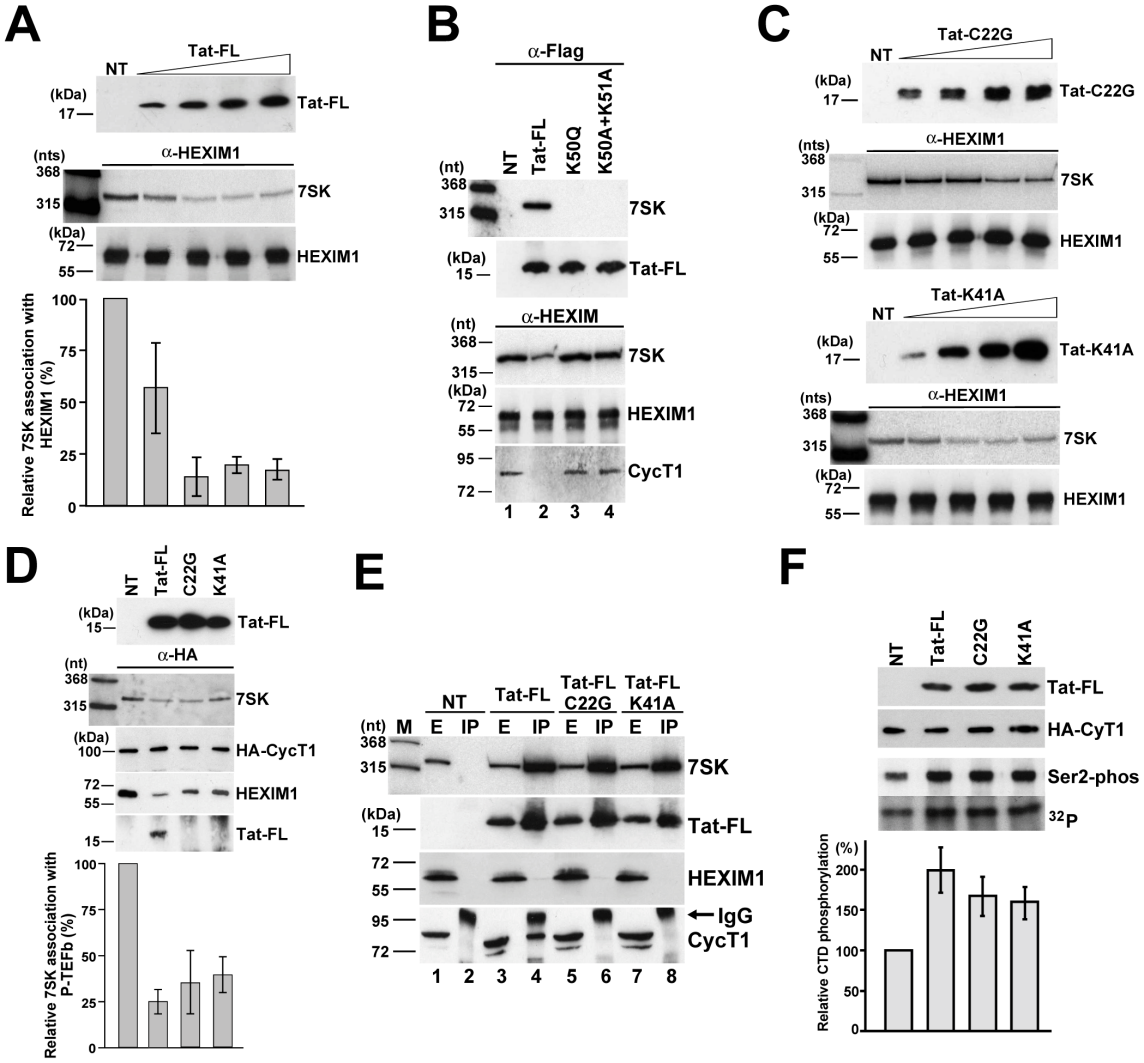
*In vivo* disruption of the 7SK/HEXIM/P-TEFb snRNP by HIV Tat. A. Tat disrupts the interaction of 7SK and HEXIM1. About 5×10^6^ HeLa cells were transfected with 0.5, 1.5, 2.5 or 3.5 µg of pTat-FL. After 48h of incubation, cell extracts were prepared, HEXIM1 was immunoprecipitated and association of 7SK snRNA was measured by Northern blotting. NT, control IP from non-transfected cells. B. The TAR RNA-binding capacity of Tat is essential for 7SK binding and for disruption of the 7SK/HEXIM/P-TEFb snRNP. Transiently expressed wild-type and mutant (K50Q and K50A+K51A) Tat-FL proteins as well as endogenous HEXIM1 were immunoprecipitated and co-purification of endogenous 7SK snRNA and CycT1 was monitored. C. Transiently expressed mutant C22G and K41A Tat proteins lacking CycT1-binding ability can disrupt the *in vivo* interaction of HEXIM1 and 7SK. (For other details, see the captures to panel A) D. Tat disrupts 7SK/HEXIM/P-TEFb independently of its CycT1-binding capacity. Wild-type and mutant (C22G and K41A) Tat-FL proteins were expressed in HeLa G3H cells stably expressing HA-CycT1. Association of HA-CycT1 with 7SK, HEXIM1 and Tat-FL proteins was monitored by co-IP. E. Tat and HEXIM1 bind to 7SK in a mutually exclusive manner. From extracts (E) prepared from HeLa cells non-transfected (NT) or transfected with the pTat-FL, pTat-FL(C22G) or pTat-FL(K41A) expression plasmids the accumulating Tat-FL proteins were immunoprecipitated (IP). Distribution of 7SK snRNA and Tat-FL, HEXIM1 and CycT1 proteins was monitored with RNase A/T1 mapping and Western blot analysis. F. Expression of Tat-FL, Tat-FL(C22G) and Tat-FL(K41A) increases the cellular level of active P-TEFb. From extracts of G3H cells expressing Tat-FL proteins, the HA-tagged P-TEFb was immobilized on beads saturated with anti-HA antibody and incubated with a recombinant GST-CTD protein and [γ-^32^P]ATP. Distribution of HA-CycT1 and Tat-FL and phosphorylation of GST-CTD at serine 2 were monitored by Western blot analysis. CTD phosphorylation was quantified by PhosphorImager.

Demonstration that HIV Tat binds to the distal HEXIM-binding site of human 7SK snRNA is consistent with the idea that Tat competes with HEXIM for 7SK binding [37]. Indeed, replacement of one copy of HEXIM with Tat would be expected to fully disrupt the 7SK-HEXIM interaction, since *in vivo* recruitment of a HEXIM-dimer requires both HEXIM-binding sites of the 7SK snRNA (Figure 5). To confirm this hypothesis, increasing amounts of Tat-FL was transiently expressed in HeLa cells (Figure 6A). After IP of equal amounts of HEXIM1, co-precipitation of 7SK was monitored by Northern blot analysis followed by PhosphorImager quantification. The ectopically expressed Tat-FL efficiently disrupted the interaction of the endogenous HEXIM1 with 7SK snRNA. Since 7SK and HEXIM can form a stable complex even in the absence of P-TEFb or other components of the 7SK snRNP [26], we assumed that the observed Tat-mediated disruption of the 7SK-HEXIM1 interaction was due to direct competition of Tat and HEXIM1 for 7SK binding. To confirm this conclusion, mutant Tat-FL proteins, Tat-FL(K50Q) and Tat-FL(K50A+K51A) [42], lacking TAR RNA-binding capacity were transiently expressed in HeLa cells (Figure 6B). Co-IP experiments demonstrated that in contrast to the wild-type Tat (lane 2), the mutant Tat proteins failed to bind 7SK snRNA and to disrupt the interaction of HEXIM1 with 7SK and CycT1 (lanes 3 and 4).

The experiments presented thus far demonstrate that the RNA-binding activity of Tat is crucial for disruption of the 7SK/HEXIM/P-TEFb snRNP. However, given that Tat can specifically interact also with CycT1, it remains possible that binding of Tat to the CycT1 subunit of the 7SK/HEXIM/P-TEFb snRNP may also contribute to the disassembly of this particle [38]. To test this possibility, we assayed the 7SK-HEXIM1 interaction in HeLa cells expressing increasing amounts of mutant Tat-FL(C22G) and Tat-FL(K41A) proteins lacking CycT1-binding ability (Figure 6C). Similarly to the wild-type Tat (see Figure 6A), both mutant Tat proteins reduced the association of HeLa 7SK snRNA with HEXIM1 in a concentration-dependent fashion. Next, the mutant Tat-FL(C22G) and Tat-FL(K41A) proteins missing CycT1-binding capacity were transiently expressed in HeLa G3H cells which stably expressed HA-CycT1 [54], [55] (Figure 6D). After IP of comparable amounts HA-CycT1, co-precipitation of the endogenous 7SK snRNA and HEXIM1 as well as the ectopically expressed Tat-FL proteins was monitored. As expected, HA-CycT1 interacted with the wild-type Tat-FL, but it failed to bind to the mutant Tat-FL(C22G) and Tat-FL(K41A) proteins. More importantly, expression of the wild-type and mutant Tat proteins largely reduced the association of HA-P-TEFb with 7SK and HEXIM1. We believe that the intact 7SK/HEXIM/P-TEFb (about 25–35%) that remained in the extracts likely derived from non-transfected cells. Thus, we concluded that Tat can promote the *in vivo* disassembly of 7SK/HEXIM/P-TEFb independently of its CycT1-binding capacity.

The concept that Tat efficiently competes with HEXIM for 7SK binding, implies that Tat and HEXIM bind to the 7SK snRNA in a mutually exclusive manner. To test this, the wild-type and mutant (C22G and K41A) Tat-FL proteins were expressed in HeLa cells (Figure 6E). Co-IP experiments showed that all Tat-FL proteins efficiently associated with HeLa 7SK snRNA but showed no detectable interaction with HEXIM1, demonstrating that Tat and HEXIM interact with 7SK snRNA in a mutually exclusive manner (lanes 4, 6 and 8). As expected, the mutant Tat proteins failed to associate with CycT1 (lanes 6 and 8), but the wild-type Tat-FL interacted with CycT1 (lane 4). Apparently, the wild-type Tat associates with endogenous CycT1 predominantly in a 7SK-independent manner [42], [56], but a minor fraction of Tat may also be involved in formation of the recently reported 7SK/Tat/P-TEFb complex [42].

To further confirm that the CycT1-binding activity of Tat is dispensable for disruption of 7SK/HEXIM/P-TEFb, we assayed whether expression of Tat-FL(C22G) and Tat-FL(K41A) could increase the nuclear level of active P-TEFb. The control and mutant Tat-FL proteins were transiently expressed in G3H cells. Upon IP with an anti-HA antibody, the beads with immobilized HA-P-TEFb were incubated with a recombinant GST-CTD protein carrying 48 C-terminal copies of the consensus CTD repeat (YSPTSPS) in the presence of [γ-^32^P]ATP (Figure 6D). The phosphorylated GST-CTD was fractionated on a SDS-polyacrylamide gel and the specificity of the phosphorylation reaction was confirmed by Western blotting with an antibody specific for serine 2-phosphorylated CTD. The phosphorylation level of GST-CTD was determined by PhosphorImager quantification. Although comparable amounts of HA-P-TEFb (HA-CycT1) were attached to the beads, the extracts accumulating the wild-type and mutant Tat-FL proteins showed about 1.5 to 2-fold higher CTD phoshorylation activity compared to the non-transfected control extract. Given that in HeLa cells about 50% of P-TEFb is sequestered into 7SK/HEXIM/P-TEFb, we concluded that expression of the wild-type and mutant Tat proteins efficiently mobilized the nuclear pool of inactive P-TEFb. These results further corroborated the notion that the CycT1-binding capacity of Tat is not required for the Tat-induced disruption of the 7SK/HEXIM/P-TEFb snRNP and for increasing the nuclear level of active P-TEFb.

### Tat does not interact with HeLa 7SK/hnRNP particles

**Figure 7:**
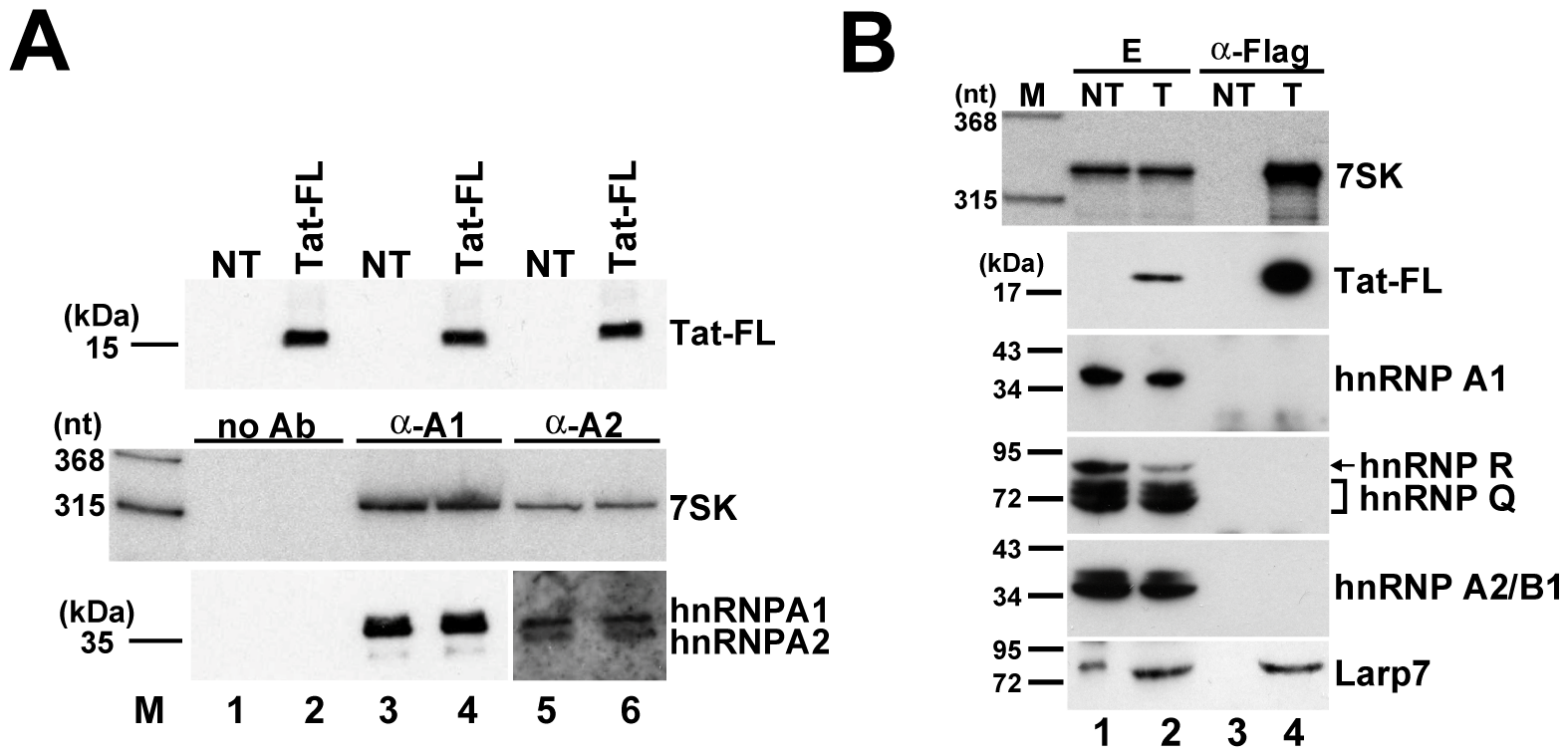
The 7SK/Tat snRNP does not interact with hnRNP proteins. A. Expression of Tat has no effect on the interaction of 7SK and hnRNP proteins. HnRNP A1 and A2/B1 were immunoprecipitated from extracts prepared from HeLa cells expressing or not expressing (NT) Tat-FL. Co-purification of 7SK was assayed by Northern blotting. Lanes no Ab, control IPs without antibody. B. The 7SK/Tat snRNP lacks hnRNP proteins, but associates with Larp7. Transiently expressed Tat-FL was immunoprecipitated from extracts (E) prepared from transfected (T) or non-transfected (NT) HeLa cells. Co-IP of 7SK snRNA and hnRNP and Larp7 proteins was assayed.

In HeLa cells, a fraction of 7SK snRNA is associated with hnRNP proteins, mainly A1, A2/B1, R and Q [44], [57], [58]. To exclude the formal possibility that the newly detected 7SK/Tat RNP or at least a fraction of 7SK/Tat derived from the hnRNP-associated pool of 7SK, we tested the effect of Tat expression on HeLa 7SK/hnRNP complexes (Figure 7A). Transient expression of wild-type Tat-FL had no detectable effect on the level of association of hnRNP A1 and A2/B1 with 7SK snRNA (lanes 4 and 6), demonstrating that the hnRNP-associated fraction of 7SK is not available for *in vivo* interaction with HIV Tat.

The hnRNP proteins associate with 7SK snRNA after its stress-induced release from the 7SK/HEXIM/P-TEFb snRNP [44], [57], [58]. We tested whether after Tat-mediated disruption of 7SK/HEXIM/P-TEFb, the resulting 7SK/Tat snRNP associates with hnRNP proteins (Figure 7B). Transiently expressed Tat-FL, together with 7SK snRNA, was immunoprecipitated and co-purification of hnRNP A1, A2/B1, R, Q and Larp7 was tested by Western blot analysis. Although the 7SK/Tat-FL snRNP interacted with Larp7, none of the tested hnRNP proteins were detected in the pellet, demonstrating that the newly described 7SK/Tat snRNP contains no hnRNP proteins.

## Discussion

HIV Tat is an unconventional transcriptional activator that, instead of targeting DNA promoter elements, recruits P-TEFb for HIV transcription through binding to a *cis*-acting RNA enhancer, the TAR RNA. Besides tethering P-TEFb to TAR, Tat also promotes the release of active P-TEFb from the 7SK/HEXIM/P-TEFb negative transcriptional regulatory snRNP [37], [38]. In this work, we demonstrate that Tat binds specifically to one of the two HEXIM-binding sites of human 7SK snRNA to displace the HEXIM homodimer on 7SK and to disrupt the 7SK/HEXIM/P-TEFb snRNP.

### The 5′ hairpin of human 7SK snRNA contains two HEXIM-binding motifs

Although all previous studies agreed that HEXIM binds to the 5′ hairpin of 7SK, its precise docking site remained uncertain [26], [27], [53]. Here, we demonstrated that the 5′ hairpin of human 7SK snRNA contains two distinct HEXIM-binding sites which are confined to the A34-C45/G64-A77 distal (DHBS) and the C12-A27/U84-U95 proximal (PHBS) segments (Figure 4). Under *in vitro* conditions, each HEXIM-binding motif of 7SK specifically and independently interacts with one HEXIM molecule.

Both HEXIM-binding sites of the human 7SK snRNA contain a stem-bulge-stem core motif (C37–C45/G64–G70 and G18-A27/U84-C90) which are highly reminiscent of the consensus structure of the minimal Tat-binding element of HIV TAR (Figure 2B and 2C). Consistent with this, HIV Tat and HEXIM proteins carry similar, positively charged, arginine-rich RNA-binding motifs which are essential for interaction with TAR and 7SK RNAs (Figure S1). These observations, together with the finding that Tat binds to the distal HEXIM-binding site of 7SK (Figure 3), strongly suggest that HEXIM and Tat use similar structural and molecular principles to recognize 7SK and TAR RNAs. Along this line of speculation, given that Tat can bind to the HEXIM-binding site of 7SK, it seems to be logical to hypothesize that HEXIM can interact with the Tat-binding site of HIV TAR [38]. However, contrary to our repeated efforts, we failed to detect a specific interaction between HEXIM and TAR (our unpublished data). The lack of TAR-binding ability of HEXIM could be explained by the observations that binding of HEXIM to 7SK snRNA, besides the TAR-like C37–C45/G64–G70 and G18-A27/U84-C90 stem-bulge-stem core motifs, also requires the adjacent A34-C36/C71-A77 and C12-U17/U1–U95 proximal sequences (Figure 4) [26]. Thus, HEXIM seems to form a more intricate interaction with 7SK snRNA than it has been reported for the Tat-TAR complex. The Tat-like RNA-binding motifs of HEXIM proteins are N-terminally extended by highly conserved positively charged regions which may contribute to the specificity of the HEXIM-7SK interaction (Figure S1). Apparently, understanding of the accurate molecular and structural background of the interaction of HEXIM and 7SK snRNA requires further efforts.

### The two HEXIM-binding sites of 7SK function in an interdependent manner in vivo

A key achievement of the current study is the demonstration that in living cells, assembly of the 7SK/HEXIM/P-TEFb snRNP requires both the distal and proximal HEXIM-binding sites of 7SK, since they recruit a homodimer of HEXIM in an strictly interdependent fashion (Figure 5). Most probably, concerted binding of two HEXIM molecules increases the 7SK-binding affinity of the tethered HEXIM-dimer. For example, structural rearrangements of HEXIM induced by dimerization may promote formation of additional molecular contacts with 7SK snRNA [59]. Interestingly, the 5′ hairpins of non-vertebrate 7SK snRNAs seem to carry only one HEXIM-docking site, suggesting that these RNAs interact with one copy of HEXIM and P-TEFb (Figure 2C). Acquisition of a second HEXIM-binding site that occurred probably through sequence duplication at the early stage of vertebrate evolution seems to be advantageous for P-TEFb regulation, since a single 7SK/HEXIM/P-TEFb dissociation event can mobilize two active P-TEFb molecules.

### Tat binds to the distal HEXIM-binding motif of human 7SK snRNA

Transiently expressed HIV Tat specifically and efficiently interacts with the endogenous human 7SK snRNA, indicating that the 7SK transcriptional regulatory snRNA is the major RNA target of Tat in the host cell (Figure 1). Tat binds to the evolutionarily conserved C37–C45/G64–G70 internal stem-loop-stem region of the 5′ hairpin of human 7SK snRNA (Figure 3). The newly identified Tat-binding motif of 7SK perfectly conforms to the consensus structure of the Tat-binding motif of HIV TAR and it is part of the distal HEXIM-binding site of 7SK.

Most of the available data are consistent with the idea that HIV Tat competes with HEXIM1 for 7SK snRNA binding to promote disassembly of the 7SK/HEXIM/P-TEFb snRNP and to increase the nuclear pool of active P-TEFb. Previous *in vitro* reconstitution experiments showed that Tat could disrupt pre-assembled 7SK/HEXIM/P-TEFb complexes, resulting in stable 7SK/Tat complex and free HEXIM and P-TEFb [38]. Unfortunately, because of the high tendency of recombinant Tat protein for oxidation and aggregate formation [48], [60], *in vitro* competition experiments require the usage of a great eccess of recombinant Tat, making it difficult to measure and compare the correct *in vitro* 7SK-binding affinities of Tat and HEXIM [38, our unpublished data]. Nevertheless, the *in vivo* competition experiments presented in this study confirmed that Tat efficiently disrupts the association of HEXIM1 and 7SK snRNA upon formation of 7SK/Tat snRNP (Figure 6). Given that both HEXIM-binding sites of 7SK are necessary for *in vivo* recruitment of a HEXIM homodimer, disruption of the 7SK-HEXIM interaction at the distal HEXIM-binding site by docking Tat is expected to release both copies of HEXIM (Figure 5). Providing strong support to this idea, Tat and HEXIM bind to 7SK in a mutually exclusive manner, neither the 7SK/Tat RNP contains HEXIM nor the 7SK/HEXIM complex associates with Tat (Figure 6) [42].

Binding of HEXIM to 7SK is the first and decisive step in the assembly of the 7SK/HEXIM/P-TEFb negative transcriptional regulatory snRNP, because association of HEXIM and P-TEFb is strictly 7SK-dependent [11], [27], [28]. Free HEXIM cannot bind to CycT1, because its negatively charged CycT1-binding domain forms intramolecular interactions with the adjacent positively charged 7SK-binding motif [28]. Docking 7SK disrupts this autoinhibitory interaction and turns HEXIM into an active conformation ready to bind and inhibit P-TEFb. Thus, disruption of the interaction of 7SK and HEXIM by Tat is predicted to mobilize the 7SK/HEXIM-associated inactive pool of P-TEFb by triggering its release from HEXIM1. In an alternative model, Tat has been proposed to mediate 7SK/HEXIM/P-TEFb disruption through competing with HEXIM for binding to CycT1 [37]. Arguing against this scenario, amino acid alterations which abolished the interaction of Tat with CycT1 only slightly reduced the ability of the mutant Tat proteins to disrupt 7SK/HEXIM/P-TEFb and to increase P-TEFb activity *in vivo* (Figures 6D and 6F). In contrast, disruption of the 7SK-binding capacity of Tat fully abolished its ability to replace HEXIM1 and to disrupt 7SK/HEXIM/P-TEFb (Figure 6B). Thus, although it remains possible that the CycT1-binding activity of Tat slightly contributes to the Tat-mediated disassembly of 7SK/HEXIM/P-TEFb, our results indicate that the P-TEFb mobilization capacity of Tat depends mostly, if not exclusively, on its 7SK-binding activity.

During revision of the current manuscript, the human 7SK snRNA has been reported to form a stable complex with Tat and P-TEFb [42]. Since 7SK can bind only one molecule of Tat and consequently, one copy of P-TEFb (Figure 3), Tat-induced disruption of 7SK/HEXIM/P-TEFb is expected to release at least half of the associated P-TEFb in the form of free P-TEFb. However, we and others observed that Tat expression converted the nuclear pool of inactive 7SK/HEXIM1/P-TEFb into free, active P-TEFb with a very high (75–95%) efficiency, suggesting that the newly described 7SK/Tat/P-TEFb accumulates at low levels (Figure 6D) [37], [38]. Nevertheless, the functional significance of the novel 7SK/Tat/P-TEFb RNP played in HIV expression remains to be established.

Demonstration that the distal HEXIM-binding site of the human 7SK snRNA encompasses a perfect Tat-binding motif has an important biomedical impact. Targeting the Tat-binding site of HIV TAR RNA with small-molecule drugs to block Tat-mediated transactivation is a very attractive approach for anti-viral therapy [61]. However, potential anti-HIV drugs with strong TAR-binding capacity are expected to interact also with the distal HEXIM-binding motif of 7SK and therefore, to promote the disassembly of 7SK/HEXIM/P-TEFb that shifts the P-TEFb equilibrium toward the active form. Since increased P-TEFb activity may have deleterious effects [16], [62], therapeutic targeting of HIV TAR requires the design of ligands which are highly specific for the TAR RNA. So, our results suggest that drug-mediated therapeutic inhibition of Tat-TAR interaction requires more precautions than anticipated before.

## Materials and Methods

### General procedures

Unless stated otherwise, all techniques used for manipulation of DNA, RNA oligonucleotides and proteins were performed according to standard laboratory procedures. The identity of all plasmid constructs was verified by sequence analysis. Human HeLa and G3H cells, the latter was provided by Dr Q. Zhou [63], were grown in Dulbecco's modified Eagle medium supplemented with 10% fetal calf serum (Invitrogen). Expression plasmids were introduced into HeLa and G3H cells by using the FuGENE transfection reagent (Roche).

### Plasmid construction

Construction of the p7SK, p5′HP and pHA-HEXIM1 expression plasmids has been described [26]. The pTat-FL, pTat-FL(C22G), pTat-FL(K41A), pTat-FL(K50Q) and pTat-FL(K50A+K51A) plasmids have been provided by Dr M. Benkirane [42], [64]. The p7SKdm, p7SKpm, p5′HPdm, p5′HPpm, p5′HPpm+dm, p5′HP*(U40–U41)*, p5′HP*(U72-C75)*, p5′HP*(U68-G70)*, p5′HP*(G64-U68)*, p5′HP*(U40)*, p5′HP*(A43)* and p5′HP*(G42-A43)* 7SK expression plasmids were generated by PCR-mediated mutagenesis using p7SK and p5′HP as templates.

### RNA analysis

RNA isolation from HeLa and G3H cells and cell extracts has been described [26]. RNAs co-immunoprecipitated with Tat-FL were 3′ end-labeled with [5′-^32^P]pCp and T4 RNA ligase before fractionation on a 6% sequencing gel. After elution from the gel, the labeled 7SK snRNA was partially digested with RNase T1 in 25 mM Na-citrate, pH 5.0, and 7 M urea at 55°C. Partial RNA hydrolysis was performed in deionized formamide containing 0.4 mM MgCl_2_ at 100°C. For Northern blot analysis, RNAs were size-fractionated on a 6% denaturing gel and electroblotted onto a Hybond-N nylon membrane (Amersham Biosciences). The filters were probed with labeled oligonucleotides complementary to the human 7SK snRNA from U92 to G111, from G272 to C291 or from C48 to C67. To generate sequence-specific antisense RNA probes for mapping of 7SK, 7SKpm, and 7SKdm RNAs, the corresponding expression plasmids were linearized with *Pst*I and used as templates for *in vitro* transcription with T7 RNA polymerase. RNase A/T1 protection analysis has been described [26].

### Complex formation and bandshift assays

To generate template DNAs for *in vitro* synthesis of internally ^32^P-labeled 5′HP, Dist, 5′HPdm, 5′HPpm and 5′HPdm+pm probe RNAs, the corresponding fragments of the p5′HP, p5′HPdm, p5′HPpm and p5′HPdm+pm plasmids were amplified with appropriate oligonucleotides which also incorporated the T7 promoter. Template DNAs for synthesis of Prox, DHBS, PHBS and 5′HPsyn were obtained by annealing of appropriate synthetic oligonucleotides followed by cloning into the pBluescribe plasmid. Recombinant HEXIM1 was purified as described [26]. Tat(38–72) peptide was synthesized by PolyPeptide Group [51]. RNA-protein complexes were formed in 50 mM Tris-HCl, pH 8.0, 100 mM NaCl, 14.4 mM 2-mercaptoethanol in the presence of 20 ng/µl of E. Coli tRNA at RT. The complexes were analyzed on 4% or 5% polyacrylamide gels (29∶1 acrylamide∶*bis*-acrylamide) containing 2.5% glycerol in 0.5× TBE.

### In vitro CTD phosphorylation

Human G3H cells transiently expressing wild-type or mutant (C22G and K41A) Tat-FL proteins were lysed in buffer A (20 mM HEPES, pH 7.9, 150 mM NaCl, 1.5 mM MgCl_2_, 1 mM DTT, 0.5 mM EDTA, 0.05% Nonidet P40) supplemented with 10 U/ml of RNasin (Promega) and protease inhibitor cocktail (Roche). After centrifugation at 10,000× g for 10 min, the extracts were incubated with anti-HA-agarose beads (Sigma) for 1 hour. The beads were washed five times in buffer A, resuspended in 100 µl of buffer A and incubated with 2 µg of recombinant GST-CTD containing 48 consensus CTD repeats (YSPTSPS) and 10 µCi of [γ-^32^P]ATP (6000 Ci/mmol) at 30°C. Phosphorylation of GST-CTD was measured by PhosphorImager quantification after fractionation on 8% SDS/polyacrylamide gel.

## Supporting Information

Figure S1 Comparison of the positively charged RNA-binding motifs of HIV Tat and HEXIM proteins. HIV Tat (AAC29057), human (NM_006460; NM_144608), *Xenopus laevis* (NP_001090038), *Salmo salar* (NP_001133431), *Danio rerio* (NP_001091859), *Ciona intestinalis* (XP_002128947), *Ixodes scapularis* (XP_002408010), *Strongylocentrotus purpuratus* (XP_792438) and *Nematostella vectensis* (XP_001636835) HEXIM proteins were obtained from the GenBank. The regions conserved in Tat and HEXIM proteins are boxed.
(0.17 MB TIF)
