# Supplementary figures and images for: Controlling Cellular P-TEFb Activity by the HIV-1 Transcriptional Transactivator Tat
Source: PLoS Pathog. 2010 Oct 14;6(10):e1001152. doi: 10.1371/journal.ppat.1001152 (PMC2954905; doi:10.1371/journal.ppat.1001152)

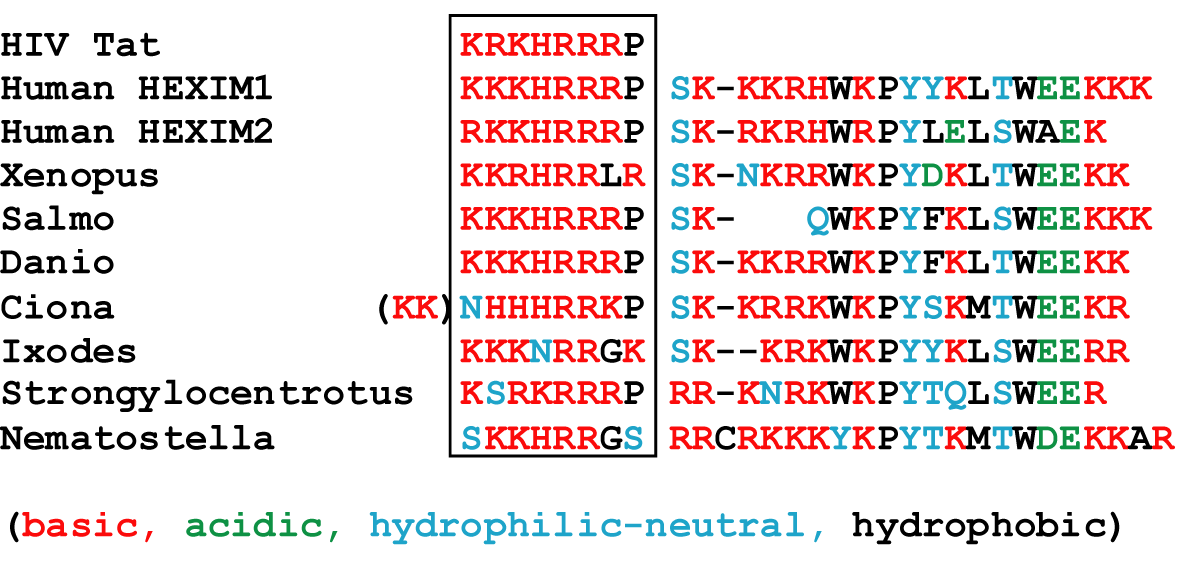

Supplement: Figure S1 — Comparison of the positively charged RNA-binding motifs of HIV Tat and HEXIM proteins. HIV Tat (AAC29057), human (NM_006460; NM_144608), Xenopus laevis (NP_001090038), Salmo salar (NP_001133431), Danio rerio (NP_001091859), Ciona intestinalis (XP_002128947), Ixodes scapularis (XP_002408010), Strongylocentrotus purpuratus (XP_792438) and Nematostella vectensis (XP_001636835) HEXIM proteins were obtained from the GenBank. The regions conserved in Tat and HEXIM proteins are boxed. (0.17 MB TIF) [file ppat.1001152.s001.tif]
